# Direct observation of small molecule activator binding to single PR65 protein

**DOI:** 10.1038/s44328-024-00018-7

**Published:** 2025-01-16

**Authors:** Annie Yang-Schulz, Maria Zacharopoulou, Sema Zeynep Yilmaz, Anupam Banerjee, Satyaki Saha, Daniel Nietlispach, Michael Ohlmeyer, Mert Gur, Laura S. Itzhaki, Ivet Bahar, Reuven Gordon

**Affiliations:** 1 Department of Electrical Engineering, University of Victoria, Victoria, BC V8W 3P6 Canada; 2 Department of Pharmacology, University of Cambridge, Tennis Court Road, Cambridge, CB2 1PD UK; 3 Department of Mechanical Engineering, Istanbul Technical University, Istanbul, 34437 Turkey; 4 Laufer Center for Physical and Quantitative Biology, Stony Brook University, Stony Brook, NY 11794 USA; 5 Department of Biochemistry and Cell Biology, School of Medicine, Stony Brook University, Stony Brook, NY 11794 USA; 6 Department of Biochemistry, University of Cambridge, Tennis Court Road, Cambridge, CB2 1QW UK; 7 Atux Iskay LLC, Plainsboro, New Jersey, NJ 08536 USA; 8 Department of Computational and Systems Biology, School of Medicine, University of Pittsburgh, Pittsburgh, PA 15213 USA

**Keywords:** Techniques and instrumentation, Cancer, Pharmacology

## Abstract

The reactivation of heterotrimeric protein phosphatase 2A (PP2A) through small molecule activators is of interest to therapeutic intervention due to its dysregulation, which is linked to chronic conditions. This study focuses on the PP2A scaffold subunit PR65 and a small molecule activator, ATUX-8385, designed to bind directly to this subunit. Using a label-free single-molecule approach with nanoaperture optical tweezers (NOT), we quantify its binding, obtaining a dissociation constant of 13.6 ± 2.5 μM, consistent with ensemble fluorescence anisotropy results but challenging to achieve with other methods due to low affinity. Single-molecule NOT measurements reveal that binding increases optical scattering, indicating PR65 elongation. This interpretation is supported by all-atom molecular dynamics simulations showing PR65 adopts more extended conformations upon binding. This work highlights NOT’s utility in quantifying binding kinetics and structural impact, offering insights valuable for drug discovery.

## Introduction

PR65, the scaffolding subunit of the serine/threonine protein phosphatase 2A (PP2A) family, provides a platform for the assembly of this heterotrimeric complex^1^. PP2A plays a critical role in regulating cell proliferation, maintaining cellular homeostasis and orchestrating cellular signaling pathways^2,3^. Dysregulation of PP2A has been linked to various cancers and chronic conditions such as Alzheimer’s disease and chronic obstructive pulmonary disease, thus presenting itself as a target for therapeutic intervention^4–8^. Reactivation of PP2A through a small-molecule activator of PP2A (SMAP), such as ATUX-8385, has shown to decrease hepatoblastoma and neuroblastoma cell proliferation in vitro and in animal models^9^. The aforementioned SMAPs are of the tricyclic sulfonamide class and bind directly to PR65, the subunit A of the trimeric PP2A. PR65 itself comprises of 15 *α*-helical HEAT repeats. The HEAT repeats of PR65 form an elongated curved structure, with one helix from each repeating forming the outer, convex surface, and the other forming the inner, concave surface onto which the regulatory (B) and catalytic (C) subunits assemble. These SMAPs have been reported to drive conformation changes that subsequently promote heterotrimer formation^10^. Mechanistically, the ligand-induced conformation change is analogous to mechanical extension or compression^10,11^. The structural impacts of ATUX-8385 on PR65 upon binding allow for a deeper understanding of the activation mechanism. Furthermore, for safety, efficacy and duration of response, it is important to understand the binding kinetics of any small molecule drug candidate.

While there have been a variety of techniques employed to study the structural changes and binding kinetics of small molecules with proteins, here we focus on nanoaperture optical tweezers (NOTs). NOTs allow for studying single proteins and their interactions without the need for modifications, such as labels or tethers^12–14^. Here we use a double-nanohole (DNH) NOT for generating a large gradient force capable of holding onto nanoparticles^15,16^. More generally, shaped nanoapertures have been used in the optical tweezer field for the study of small biomolecules and other nanoparticles^16–27^. The first report of trapping of a single protein demonstrated the technique’s sensitivity by observing the conformational changes in bovine serum albumin (BSA) via changes in light transmission intensity as measured by an avalanche photodiode (APD)^12^. Integration of NOT with a nanopore further supports single protein trapping^25,28,29^ and has been used to probe the interaction of antibodies with immunotherapy-relevant ligands^30^.

Conformational changes were suggested from the NOT signal changes for two different types of proteins: *β*−*amylase* and heat-shock protein 90 (HSP90)^31^. Iron ion loading and unloading of a single ferritin was observed by seeing structural changes in real-time using this technique^32^. Recently, we have used NOTs to study the impact of point mutation on the conformational behavior of PR65^33^, showing correlation between the extension of the protein observed in the optical scattering signal of the NOT and those predicted from molecular dynamics (MD) simulations.

The goal of the present study is to use NOTs to probe the interaction of a single PR65 protein with the clinically-relevant experimental small molecule ATUX-8385. We performed binding assays under equilibrium conditions and observed biomolecular dynamics in real-time. By tracking ligand-induced conformational changes and subsequently discerning the optical scattering signal into binary states, bound and unbound, dissociation and association rate constants can be determined through the “bound” time and “unbound” time. This is similar to single-molecule Förster resonance energy transfer (FRET), however, the ligand concentration is not limited to the typical nanomolar range as we are not hindered by background fluorescence noise by looking directly at the polarizability change of a single protein^34^.

Previously, NOTs have been used to quantify established protein-drug interactions inhuman serum albumin with tolbutamide and with phenytoin, obtaining dissociation constants within the previously reported range of values^13^. NOTs were also used to study strong interactions where the binding was essentially irreversible on the timescale of observation, e.g., biotin with streptavidin and acetylsalicylic acid with cyclooxygenase 2^35^. Here we apply and extend the technique to a much less understood binding pair, to understand the binding kinetics and to observe the conformational changes induced upon binding.

In addition to NOTs, we use several techniques to investigate the binding characteristics and effects of SMAP ATUX-8385 to PR65. These include all-atom MD simulations, nano differential scanning fluorimetry (nanoDSF), nuclear magnetic resonance (NMR) and fluorescence polarization anisotropy. In terms of molecular simulations, we explored the effect of ATUX-8385 binding on the structure and dynamics of PR65 by docking simulations followed by microseconds long MD runs, as well as microseconds simulations for apo PR65. Our docking simulations revealed a binding region that encompasses the inner helices of the HEAT repeats 4 and 5 (4_*i*_ and 5_*i*_) and the outer helices of HEAT repeats 5 and 6 (5_*o*_ and 6_*o*_). ATUX-8385 showed stable binding at this site throughout the 704 ns long triplicate MD runs of MD simulation, totaling 2.112 μ*s* in combined trajectory length. ATUX-8385 binding stabilized an extended conformation in two runs and a compact form in a third run. The latter transitioned to the extended form towards the end of the 704 ns simulation, indicating the stabilizing effect of ATUX-8385 bound to PR65, and its tendency to favor the extended conformation. NanoDSF showed that the presence of ATUX-8385 stabilized PR65 through increase in the melting temperature. NMR confirmed the binding of ATUX-8385. Fluorescence polarization anisotropy experiments provided an ensemble dissociation constant in close agreement with the single molecule dissociation constant.

## Results

### Structural impact of ATUX-8385 binding: NOT experimental results

**Fig. 1:**
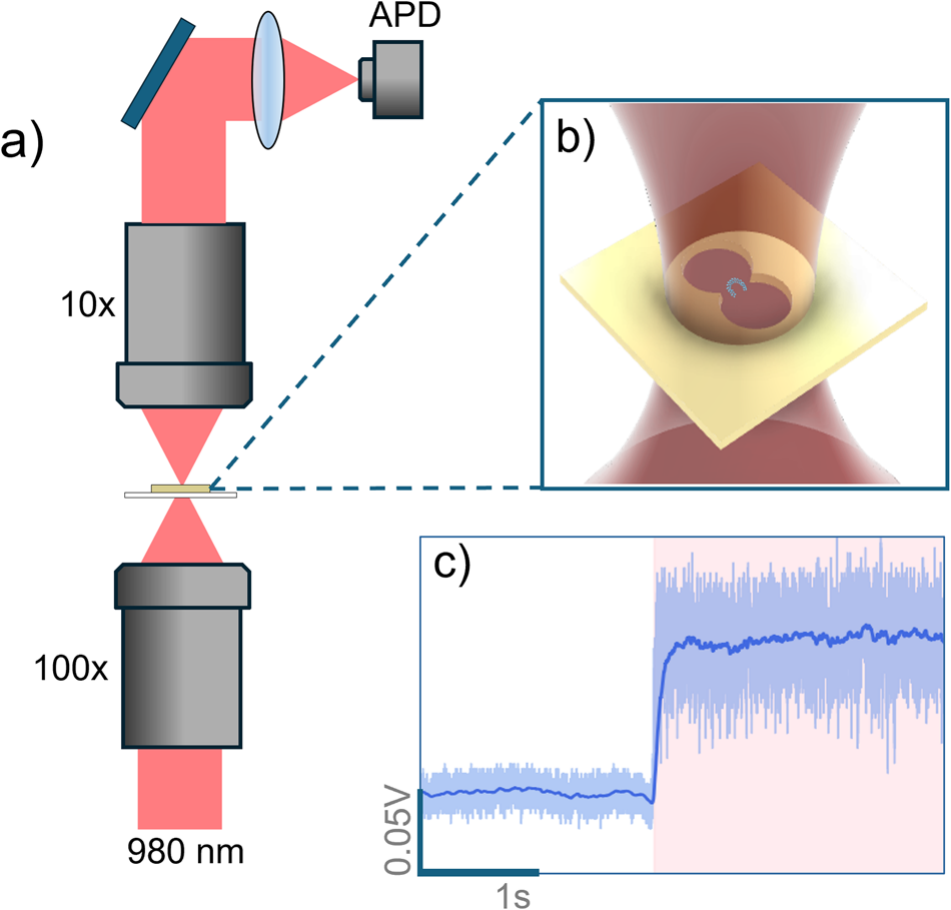
Overview of NOTs trapping. **a** Depiction of a simplified nanoaperture optical tweezer. **b** Cartoon representation, not to scale, of laser beam focused onto a DNH aperture with PR65 trapped between the DNH cusp. **c** Sample trapping signal of PR65 protein without SMAP, pink region shows when the protein has entered the trap.

A home-built optical trapping setup and DNHs fabricated colloidally were used for the experiments as shown in Fig. 1. The trapping event was characterized as the change in transmission intensity due to dielectric loading and increase in noise due to Brownian motion of the protein in the trap^16,36,37^, see Fig. 1c. A single jump in transmission level, as depicted in Fig. 1c, typically represents an individual protein trapping event unless a protein exists naturally as a homo-oligomer. In the absence of other proteins, PR65 exists as a monomer and the trapping events observed were of single proteins. By contrast, multiple protein trapping events show up clearly in the signal, as has been reported in the literature for BSA and egg white protein^38,39^. For our study, we focused on the difference between the trapped signal (transmission intensity picked up by the APD after the trapping event) of apo-PR65 in 5% DMSO and PR65 with ATUX-8385 in 5% DMSO. A total of 8 measurements were collected at 20 μM of ligand and a protein concentration of 10 μM.

**Fig. 2:**
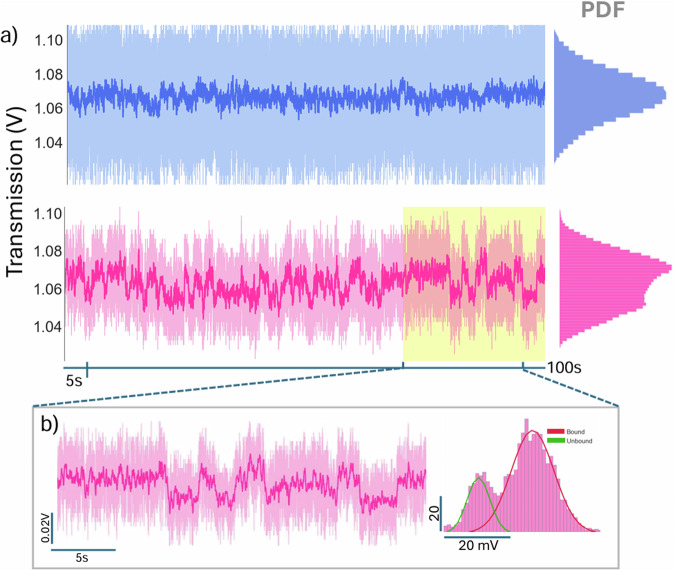
Time series signal of the trapped PR65 with (pink) and without the presence (blue) of ATUX-8385 shows the interaction between protein PR65 and SMAP. **a** The SMAP concentration used was 20 μM while the protein concentration was fixed at 10 μM. Transmission signal from different DNH is shown. Lighter signal in the background is the raw signal while the solid lines are from the 3 Hz low pass filtered signal. PDF of the filtered signals are shown to the right of the time series signal. **b** PDF of the filtered signal fit with Gaussian functions further indicates the presence of a bound and unbound state. For the bound state, the Gaussian curve occupies an area(*A*_*B*_) of 0.76 with a standard deviation(*σ*_*B*_) of 0.004 V. Likewise, the unbound state Gaussian curve has parameters: *A*_*U*_ = 0.22, and *σ*_*U*_ = 0.002.

A 100 s segment of the trapped signal of PR65 and PR65 with ATUX-8385 from separate trapping events is shown in Fig. 2. A 3 Hz low pass filter applied to the raw signal of PR65 renders a comparatively homogeneous signal when compared to the signal of PR65 in the presence of ATUX-8385. In the presence of SMAP, the intensity of transmission fluctuates between two levels, showing a conformational change of the protein^40^. The intensity changes correlate with a change in the polarizability of the protein, hence, refractive index change of the protein^16,41^. The molecular weight of PR65 is 65 kDa, which is reflected in its name. In contrast, ATUX-8385 has a molecular weight of approximately 500 Da, making it about two orders of magnitude smaller than PR65. In a previous work, the change in the standard deviation of the mean was linearly related to the protein size in the NOT system^37^. Based on this, we would expect a .7% change in standard deviation of the signal approximately with the additional mass, but in the experiment it approximately doubles (Fig. 2b). Furthermore, our all-atom MD simulations of both the apo and ATUX-8385-bound PR65, presented in the following section, show a change in the conformation upon binding, which shifts the polarizability^41^.

The probability density function (PDF) of the filtered signal is shown to the right of the time series signal. The 20 μM ATUX-8385 histogram was fit with two Gaussians for the bound and unbound states. The larger scattering signal voltage came from increased scattering (larger polarizability) and showed that binding of ATUX-8385 extends the protein. Extension of protein was also seen through the increase in the standard deviations of the Gaussian fits. Higher noise from the extended form of PR65 has been reported previously for point mutations^33^. While higher polarizability leads to a stiffer trap, it has a stronger effect on the scattering, as shown in past works^37^.

To determine whether the extended form corresponded to the bound or unbound state, the experiment was repeated at a concentration of 5 μM ATUX-8385 SMAP. At this reduced concentration, a lower frequency of the extended form was observed (see Supplementary Fig. SI-1), suggesting that the higher transmission level corresponds to the bound state. Further support for this conclusion is provided by molecular simulation results discussed in the following section.

### Molecular simulations

We performed molecular simulations to compare experimental and simulated structural impacts. Docking simulations for ATUX-8385 revealed a novel binding site. As no structural data on the binding site/poses of the SMAP molecule ATUX-8385 on PR65 exist, we conducted a series of docking simulations on PR65 structure to determine a binding pose for ATUX-8385. Based on hydroxyl radical footprinting experiments, the K194-L198 region of PR65 was identified as a putative SMAP-binding region for a different tricyclic molecule, with K194, E197, and L198 as potential interaction sites^10^. Using this information, we performed guided docking simulations of ATUX-8385 onto PR65.

First, we used the Rosetta Ligand Docking Protocol 3^42^ in the ROSIE server^43^ to find the optimal binding pose of ATUX-8385 near E197. The compact conformation of PR65 (PDB: 6NTS, chain A) was used for docking simulations. The selected conformation (Supplementary Fig. SI-2a, ATUX-8385 in yellow) binds to 4_*i*_, 5_*i*_, 5_*o*_ and 6_*o*_, and has an interaction energy of −10.5 kcal/mol. All interaction energies are calculated using PRODIGY-LIG^44^.

We then conducted additional docking simulations within 40Å; radial distance from E197, so as to explore alternative docking poses and potential binding to PR65 residues near E101, where a related SMAP, DT-061, was reported to bind and stabilize the PP2A trimer^45^. Using AutoDock Vina 1.2.0^46^, docking simulations were carried out for both compact (PDB: 6NTS, chain A) and extended (PDB: 1B3U, chain A) forms of PR65. The selected conformations of ATUX-8385 with PR65 in the compact form (Supplementary Fig. SI-2a, ATUX-8385 in red) and extended form (Supplementary Fig. SI-2b, ATUX-8385 in red) led to interaction energies of −6.6 kcal/mol and −9.0 kcal/mol. Interestingly, the same binding site (near 4_*i*_ and 5_*i*_) was shared between the compact and extended forms, while the extended form was significantly more favorable. We note that the coordinating residues D106 and R105 (among others) are adjacent to the DT-061 binding site in PP2A.

**Fig. 3:**
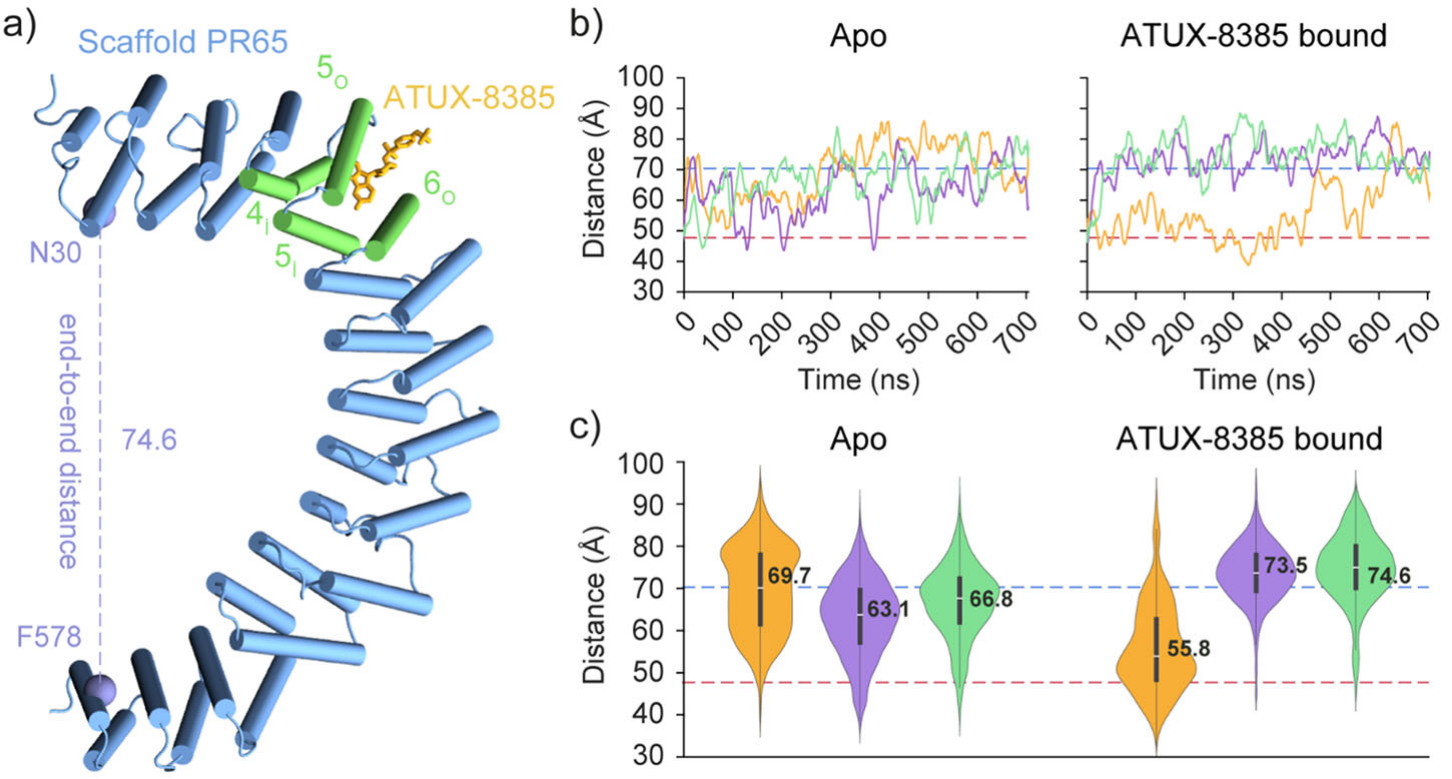
Distribution of end-to-end distances in apo and SMAP-bound PR65 from MD simulations. **a** Representative extended PR65 conformation stabilized by ATUX-8385, obtained from the third run. Distance between N30 and F578 *Cα* atoms was used to define the structural form of PR65. **b** The end-to-end distance time evolution for runs 1–3 shown in orange, purple, and green. Dashed lines depict the end-to-end distances observed in the compact (PDB: 6NTS) and extended (PDB: 1B3U) structures of PR65. **c** Violin plots depicting the distribution of end-to-end distances for PR65 during the simulations under apo and ATUX-8385-bound states. For each distribution, mean values are indicated, and standard deviations are shown with thick bars.

Since the conformation found using ROSIE was energetically more favorable, we proceeded with extensive MD simulations using this pose. MD simulations initiated from the compact PR65 structure showed that ATUX-8385 binding favored an extended conformation for PR65. Two sets of MD simulations were performed: one for apo PR65 and the other for the ATUX-8385 docked form of PR65. Each set was composed of three separate runs of 704 ns in length, totaling 4.224 μs of MD trajectory across both sets. ATUX-8385 remained in its docked position throughout all three runs, further validating this new binding site as a stable interaction point for ATUX-8385 on the PR65 monomer. ATUX-8385 binding impacted the structural preferences of PR65. While the apo PR65 runs fluctuated between compact and extended forms throughout the simulations, ATUX-8385 binding stabilized the extended PR65 form rapidly within 100 ns in two runs (Fig. 3). Interestingly, in the other run, the compact form was maintained for the first 500 ns before transitioning to an extended form. Collectively, these observations demonstrate that ATUX-8385 bound to the site formed by 4_*i*_,5_*i*_,5_*o*_ and 6_*o*_ stabilises the PR65 and favors the extended conformation. It should be noted that the fluctuations observed in the simulations are at a much faster timescale and do not correspond to the fluctuations seen in the experiment. The Boltzmann average binding affinity for this pose (Fig. 3a), calculated over 2.112 μs of MD trajectory, was −9.6 kcal/mol.

### Observation of protein binding kinetics: NOTs single molecule measurements

**Fig. 4:**
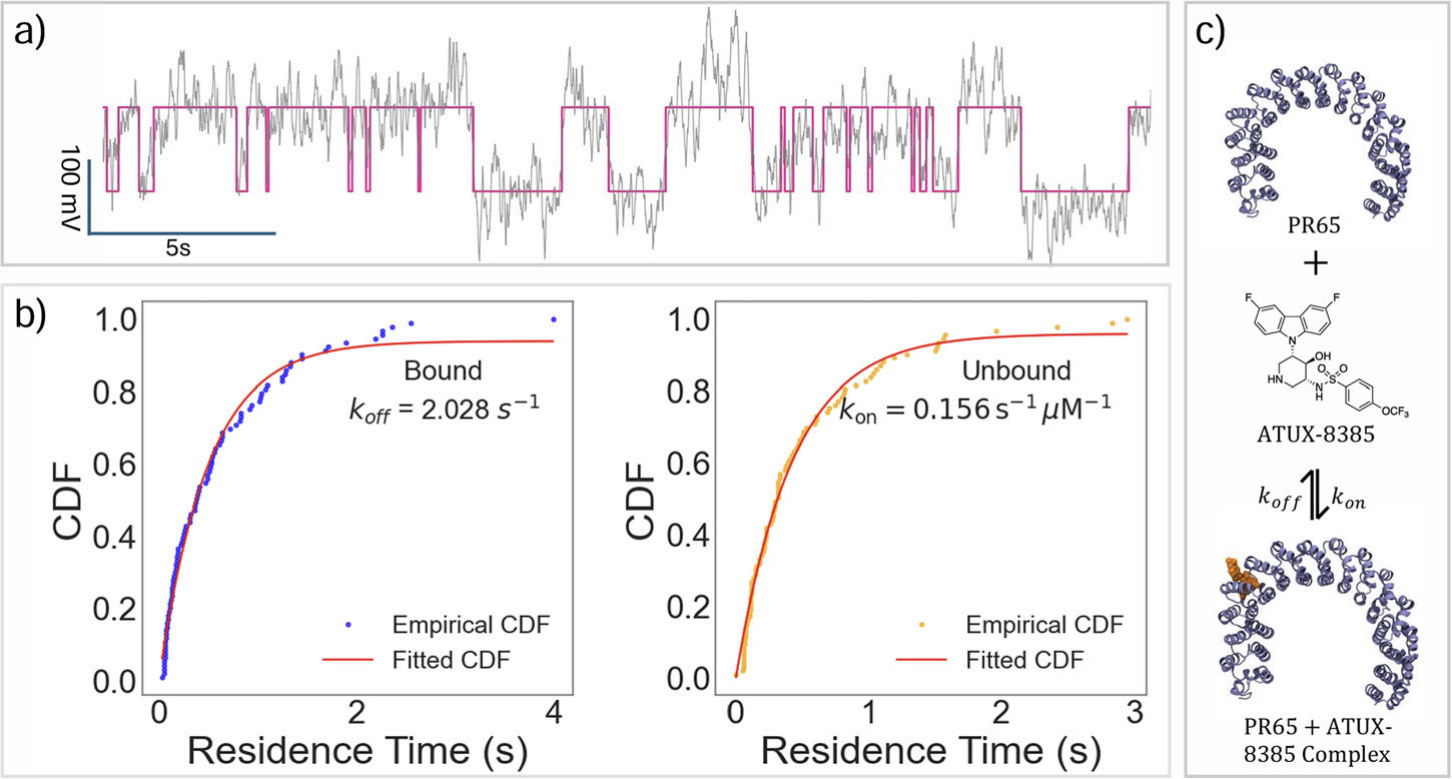
Quantification of single molecule binding kinetics. **a** Step function fit to a 30 s segment of trapped filtered signal. Higher scattering state corresponds to the protein-ligand complex. **b** CDFs of the residence time in bound/unbound states are fitted to a single exponential for the extraction of binding constants. A total of 94 events occurred in the 110 s analyzed. Coefficients of determination for bound and unbound CDF fitting are 0.9885 and 0.991 respectively. **c** Cartoon depiction of the 1:1 binding model.

The 3 Hz low-pass filter was applied to the trapped signal of a single trapping event to track the mean movement of the transmission signal, filtering out higher-frequency noise while preserving the overall trend. The choice of filtering frequency can affect the results. Testing at 10 Hz showed that filtering was insufficient, allowing spurious noise artifacts to impact the data. At 1 Hz, however, the signal was overly smoothed and distorted. We selected 3 Hz as a compromise, balancing distortion and noise artifacts. This filtered signal was then segmented into approximately 30s sections for easier processing, given the large file size. These sections were fit into step functions through a Hidden Markov Method (HMM) for identification of the two states (Fig. 4a), see Methods for details. For each trapping event, ~100 s of data was collected, segmented and analyzed. Cumulative distribution functions (CDFs) of the residence time in the bound and unbound state were fit with a single exponential function to determine association (k_on_) and dissociation rate (k_off_) constants, Fig. 4b. The good fit to a single exponential function suggests a 1:1 binding model between the protein and ligand. Individual CDF fits, association and dissociation constants can be found in Supplementary Section 3. Across the measurements made (*n* = 8), the signal showed a good consistency. Per 100 seconds, 111 ± 16 binding events occurred. The bound transmission intensity normalized to unbound transmission intensity was (101 ± 0.19)%. Table 1 summarizes the values for all measurements made at the 20 μM ATUX-8385 SMAP concentration.

**Table 1:** Summary of interaction parameters between PR65 and SMAP ATUX-8385 found from single molecule NOT measurements (*n* = 8)

*τ*_off_(*s*)	*τ*_on_(*s*)	*k*_off_ (s^−1^)	*k*_on_ (s^−1^ μM^−1^)	Δ*G*^∘^ (kcal/mol)	*K*_*D*_ (μM)
0.40 ± 0.06	0.37 ± 0.03	2.52 ± 0.42	0.19 ± 0.02	−6.72 ± 2.5	13.6 ± 2.5

### Additional characterization of SMAP binding to PR65

We used a range of biophysical techniques to confirm in vitro binding of SMAP ATUX-8385 to PR65 and to determine the binding affinity. The relatively low solubility of the small molecule (only soluble up to 100 μM in 10% DMSO, see Supplementary Fig. SI-5) precluded the use of standard techniques such as Isothermal Titration Calorimetry (ITC), but we collected orthogonal data from three different biophysical methods to confirm that ATUX-8385 binds to PR65.

**Fig. 5:**
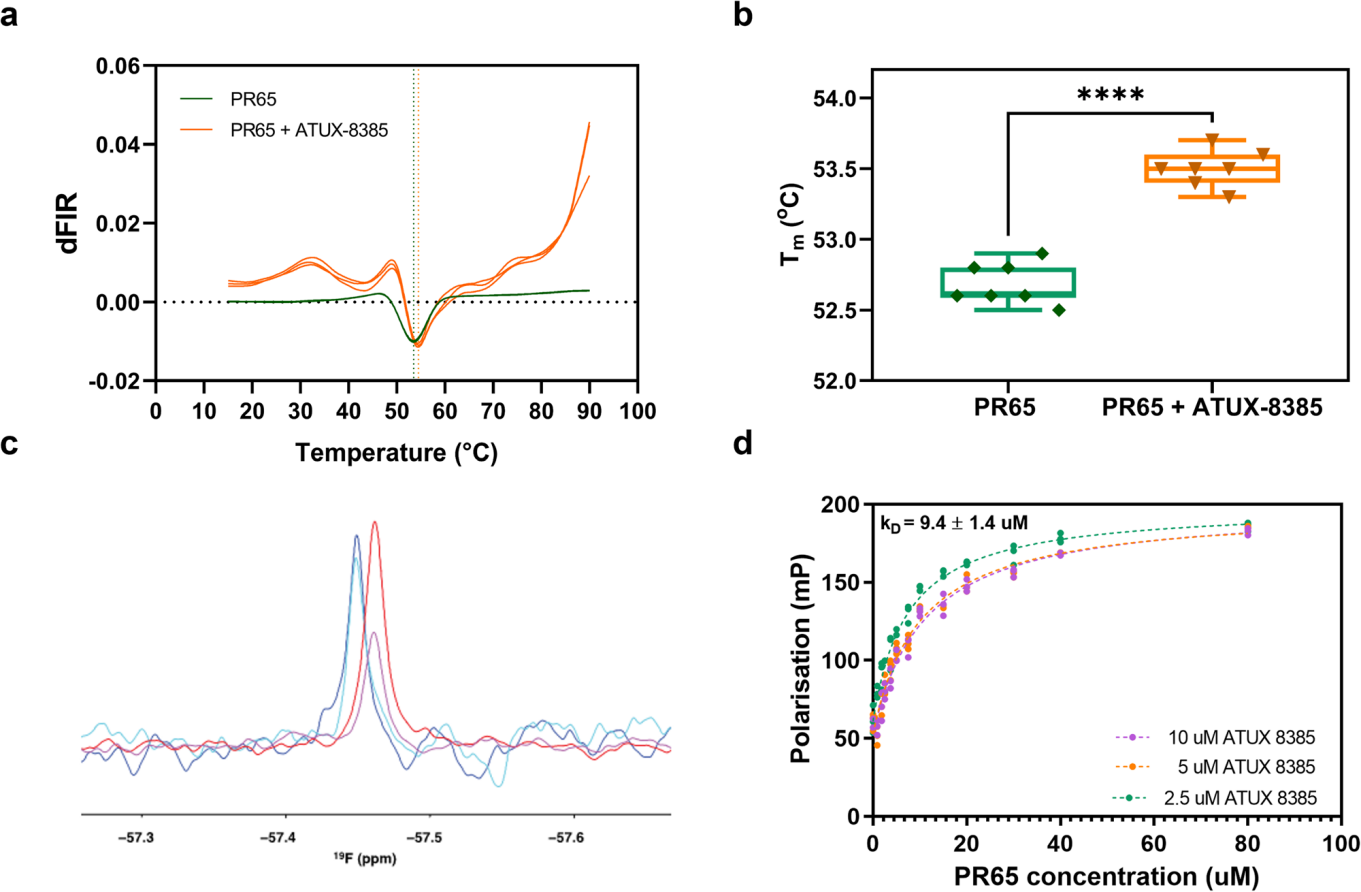
Biophysical characterization of small molecule ATUX-8385 binding with protein PR65. **a** NanoDSF traces of the thermal denaturation of PR65 in the absence (green traces) and in the presence of ATUX-8385 (orange traces). The data shown are the first derivative of the ratio of fluorescence intensities read at 350 nm and 330 nm (dFIR (350 nm/330 nm)). The global minimum corresponds to the melting temperature of the protein, *T*_*m*_. A shift towards higher *T*_*m*_ values indicates an increase in stability induced upon SMAP binding. **b** Extracted *T*_*m*_ values from the NanoDSF traces indicate an upwards shift in the presence of ATUX-8385(*T*_*m*__PR65_ = 52.7 ± 0.1 °C, *T*_*m*__PR65+ATUX-8385_ = 53.5 ± 0.1 °C, *N* = 7, *p* < 0.001 via an unpaired t-test). The NanoDSF experiments were performed with a Prometheus NanoDSF instrument (NanoTemper Technologies). 2 μM of PR65 in PBS, 2 mM DTT, was incubated either with 10% DMSO, or 100 μM ATUX-8385 in a final concentration of 10% DMSO, and thermal denaturation was performed from 20 °C to 90 °C with a 1 °C/min rate. **c** ^19^F NMR recorded at 298 K on ATUX-8385 indicates binding of the small molecule to PR65. The NMR spectra show a drop in signal intensity upon binding of the small molecule, while the change in chemical shift is small as indicative of relatively weak binding. ^19^F 1D CPMG experiments were performed for 100 μM ATUX-8385 in 10% DMSO (blue: transverse period 2 ms; cyan: transverse period 102 ms) or for 100 μM ATUX-8385 in 10% DMSO with 5 μM PR65 (red: transverse period 2 ms; magenta: transverse period 102 ms) on a Bruker Avance III 600 MHz (^19^F 564 MHz). **d** Fluorescence polarization experiments show ATUX-8385 binding on PR65 with a K_D_ in the low micromolar range. Shown are the fluorescence polarization values (mP) for 2.5 μM, 5 μM, and 10 μM ATUX-8385 upon PR65 titration, *N* = 3. One-site fitting of the data (see methods for equation) gives K_D_ = 9.4 ± 1.4 μM.

We first explored NanoDSF, a label-free, fluorescence-based technique that can be used to analyze the binding of small molecule ligands to proteins. It measures changes in the intrinsic tryptophan and tyrosine fluorescence of proteins when they undergo thermal denaturation. A shift in the melting temperature, *T*_*m*_, of the protein can be expected upon small molecule binding, as small molecules can locally or globally stabilize or destabilize the protein structure. Thermal denaturation of 2 μM PR65 was measured in 10% DMSO versus in the presence of 100 μM ATUX-8385, 10% DMSO. A positive shift in the melting temperature of PR65 was observed in the presence of ATUX-8385 (*T*_*m* PR65_ = 52.7 ± 0.1 °C, *T*_*m* PR65+ATUX_ = 53.5 ± 0.1 °C), indicating binding and slight stabilization of the protein structure (Fig. 5a, b). The relatively low observed shift in *T*_*m*_ suggested a binding affinity in the micromolar range.

To further establish the binding of ATUX-8385 to PR65, we used ^19^F-NMR, which probes Fluorine-19 on the ligand (ATUX-8385). The interaction between ATUX-8385 and PR65 was detected by measuring the signal intensity and the ^19^F relaxation rate of the small molecule in the presence and absence of PR65. The ^19^F NMR spectra were collected for 100 μM ATUX-8385 in 10% DMSO, and for 100 μM ATUX-8385 in 10% DMSO with 5 μM PR65. A singlet signal was detected at -57.46 ppm for ATUX-8385, corresponding to the -CF3 group of the molecule. Only a very small change in signal position was observed upon addition of PR65. An increase in the relaxation rate *R2* upon addition of PR65 (*R*2_*ATUX*_ = 6.93 ± 0.05, *R*2_*ATUX*+*PR*65_ = 1.85 ± 0.05), revealed an increase in the rotational correlation time, indicative of binding of ATUX-8385 to PR65 (Fig. 5c).

To quantify the binding affinity of ATUX-8385 for PR65, we exploited the fact that ATUX-8385 is a fluorescent molecule. ATUX-8385, when excited at 320 ± 10 nm wavelength light, emits at 340–550 nm, with a maximum fluorescence intensity at 370 nm (Fig. SI-5). Fluorescence polarization (FP) is, therefore, a useful technique that can be used in this system to calculate the binding affinity. FP is based on the principle that the rotational motion of a fluorescent molecule, in this case ATUX-8385, affects the polarization of emitted light. When ATUX-8385 was bound to PR65, its rotational motion was reduced, resulting in higher polarization values. FP was performed at 40 °C to match the optical trapping experiments. PR65 was titrated in concentrations ranging from 1 μM to 80 μM to samples containing 2.5 μM, 5 μM, and 10 μM of ATUX-8385 (10% DMSO) (Fig. 5d). By fitting the data to a one-site binding curve, the equilibrium dissociation constant, K_D_, was determined to be 9.4 ± 1.4 μM.

## Discussion

As PP2A holds a central role in many biological processes, there have been clinical investigations into PP2A directed therapeutics for preventing dysregulation in the enzyme. Here we explored a SMAP (ATUX-8385) of tricyclic sulfonamide class that binds directly to the scaffold unit of PP2A, PR65, which has been shown to reactivate PP2A in a previous study^9^. We employed several approaches to gain novel insights into the effects of ATUX-8385 binding on the structure and dynamics of PR65, and the binding kinetics of PR65 and ATUX-8385.

Through docking simulations, a novel binding site, comprising residues at 4_*i*_, 5_*i*_, 5_*o*_, and 6_*o*_, for ATUX-8385 was identified. Subsequent extensive MD simulations confirmed the stability of this site and demonstrated that ATUX-8385 impacts the structural dynamics of PR65. In our simulations, ATUX-8385 binding was observed to favor the extended form. Specifically, binding of ATUX-8385 stabilized the extended form in two out of three simulations and the third run converged to an extended form towards the end of the simulation.

The docking of ATUX-8385 to the 4_*i*_ and 5_*i*_ shows promise, as indicated by its vicinity to known SMAP binding site. These novel binding sites differ from previously identified sites and suggest a potentially unique mechanism of action. These findings warrant further analysis in future work, including extensive MD simulations and experimental validation, to better understand the binding dynamics and functional implications of ATUX-8385 on PR65.

We produced two separate measurements of dissociation constants in this work: single molecule and ensemble. The dissociation constant found using the NOT approach comes from a single measurement on a single protein at a single SMAP concentration, while the ensemble measurement utilized the fluorescent property of ATUX-8385 to perform fluorescence polarization experiments. The ensemble measurement required repeated measurements at different PR65 concentrations to establish the dissociation constant. The *K*_*D*_ obtained from both measurements are in good agreement with each other, and characterized ATUX-8385 as a molecule of weak affinity to bind to PR65. Using NOT, the observation of complex formation and dissociation in real time allows for determination of *k*_*on*_ and *k*_*off*_. The change in the scattering signal with complex formation is consistent with the elongation of the protein upon binding, which provides biophysical data about the interaction. This stabilization in the elongated form was also confirmed in MD simulations. Our past work quantified this elongation for various point mutations of PR65^33^.

Since the NOT technique depends on detecting light scattering changes induced by the activator upon binding, its applicability may be limited to binding pairs that produce a measurable scattering change, as not all interactions may result in detectable shifts. However, due to the same principle of the technique, the single-molecule binding assay is not restricted to the typical picomolar- or nanomolar range for techniques such as total internal reflection microscopy, smFRET. Fluorescence-based single molecule techniques are often confined to a low concentration barrier, as high concentration of fluorescent molecules results in an increase in background signal that in turn obscures binding events^34,47^. This is especially limiting as many biological processes are transient and have a dissociation constant in the micromolar range. Therefore, the NOT technique has an appropriate niche to study kinetics that can be observed on a timescale ranging from a microsecond to hundreds of seconds. For longer observation, a stable microscope setup is required to avoid drift over extended duration.

The NOT has an interesting feature of being able to tune the temperature by adjusting the trapping laser power^40,48–50^. In the present work, we limited the power to achieve temperatures near physiological values; however, future studies can be undertaken to explore the impact of temperature on the binding kinetics. We did attempt a few temperature values by varying the laser power and saw insignificant variation in the dissociation constant over 4 °C.

In this study, we explored a SMAP (ATUX-8385) of tricyclic sulfonamide class that binds to PR65 and has been shown to reactivate PP2A in a previous study aimed at cancer therapeutics^9^. We used NOT, a single molecule technique, to determine the dissociation constant of 13.6 ± 2.5 μM, agreeing with fluorescence anisotropy measurements while not requiring a titration: the experiment was performed on a single protein molecule and was highly repeatable. The NOT approach showed increased light scattering upon binding, which is consistent with an extension of the protein upon binding, as found in MD simulations. The MD simulations also elucidated the binding site. Therefore, the NOT, combined with all-atom MD simulations, provided information on the conformational changes taking place upon SMAP binding. Such mechanistic data are useful for clinical certification of SMAPs.

Label-free single particle analysis techniques are changing the landscape of biomolecule investigations, with recent successful commercialization of iScat^51,52^, nanopores^53,54^ and tethering-based force-measurement^55,56^ techniques. The NOT approach allows for valuable data extraction on small molecule binding for drug discovery applications, not only quantifying the binding affinity, but also giving insight into the impact of binding on the structure of the protein. It also has the advantage of working on a single molecule, not requiring additional steps like titrations or labeling, using less sample and allowing for tuning the local temperature at the protein by changing the laser power. Therefore, we believe that there is potential for this technique to be widely adopted in drug discovery and other research areas.

## Methods

### Protein expression and purification

GST-tagged PR65 was expressed in *E. coli* as described previously^57^. In brief, plasmid encoding PR65 was transformed into chemically competent C41 *E. coli* (Komander laboratory, MRC-LMB, Cambridge). Cultures were grown at 37 °C in 2xYT medium containing ampicillin (50 μg/ml) until an OD600 of 0.6–0.8 was reached. Protein expression was induced with 500 μM isopropyl-*β*-d-thiogalactopyranoside (IPTG) (Generon) at 25 °C overnight. Cells were harvested by centrifugation at 4000 × *g* for 10 min at 4 °C before resuspending in lysis buffer [50 mM tris-HCl (pH 7.5), 500 mM NaCl, 2 mM dithiothreitol (DTT)] supplemented with EDTA-free protease inhibitor cocktail (Sigma-Aldrich) and deoxyribonuclease (DNase) I (Sigma-Aldrich). The cells were lysed by passing the suspension two to three times through an Emulsiflex-C5 (AVESTIN) at pressures of 10,000–15,000 psi. Soluble protein was separated from cell debris and other insoluble fractions by centrifugation at 35,000 × *g* for 35 min at 4 °C. The soluble protein fraction was applied to glutathione resin [Amintra Affinity, EGTA (0.5 g/liter), 2 mM DTT], the GST tag was cleaved with thrombin, and PR65 was eluted using a gravity column. After washing the column, the protein was subsequently eluted using a 20 × column-volume salt gradient from 0 to 1 M NaCl. MonoQ fractions containing the protein were concentrated before application to a HiLoad 26/600 Superdex 200 pg (GE Healthcare) equilibrated in phosphate-buffered saline (pH 7.4) and 2 mM DTT. Samples were analyzed by SDS-PAGE (polyacrylamide gel electrophoresis) comparing the lysed, flowthrough, and eluted fractions. The identity of the protein was confirmed via MALDI mass spectrometry (Department of Chemistry, University of Cambridge).

### DNH nanoaperture fabrication

The DNH fabrication follows our past approaches^15,58^. A microscope slide with dimensions 75 × 50 × 1 mm was divided into three roughly equal pieces using a diamond scriber. The slides were sonicated in an ethanol bath for 10 min, then rinsed with acetone and isopropyl alcohol before being dried with nitrogen. To prepare the polystyrene spheres (PS) solution, 7.5 μL of ThermoFisher 300 nm PS was mixed into 1 mL of ethanol. Next, 10 μL of the PS-ethanol solution was deposited onto each slide in a zigzag pattern and left to dry overnight. The slides were plasma etched using a Harrick PDC plasma cleaner for 135 s to fuse randomly distributed dimers and reduce the cusp size as a function of etching time. A 7 nm layer of titanium was sputtered onto the slides to act as an adhesive layer for gold. Subsequently, a 70 nm layer of gold was sputtered onto the slides. To remove the PS, the slides were sonicated for 7 min. For verification purposes, the slides were then imaged using a SEM Hitachi S-4800 (Supplementary Fig. SI-6).

### Sample preparation for trapping experiments

The protein solutions comprised of phosphate-buffered saline solution with 0.01% (w/v) concentration, 2 mM of Dithiothreitol and 5% (v/v) Dimethylsulfoxide (DMSO). DMSO was used as a solvent for ATUX-8385. PR65 concentration was 10 μM while the ATUX-8385 concentration in solution was 20 μM. It is important to note that upon laser illumination, ohmic losses in the metal lead to local heating at the nanoaperture. Studies have shown that the temperature increase ranges between 0.64 K/mW to 4.25 K/mW^48,49^. The temperature increase is experimentally shown to be dependent on the intensity of the incident laser, yet independent of aperture size, DNH cusp, and laser polarization^49^. The local temperature increase at the aperture was 0.64 K/mW^39,48^. Unless otherwise stated, trapping experiments were conducted at an incident power that raised the temperature of the trapped protein to approximately 35 °C. A 50 × 2 × 0.13 mm coverslip was rinsed with acetone and ethanol, then dried with nitrogen gas. A microwell was then formed by attaching an image spacer (Grace Bio-Laboratories GBL-654008-100EA) onto the coverslip. 9.5 *μ*L of protein solution was then micro-pipetted into the microwell. The DNH slide, with the gold side facing the solution, was placed and sealed onto the image spacer.

### Trapping setup: inverted microscope

A collimated laser beam exiting a fiber passes through a linear polarizer and then a half-wave plate, which adjusts the polarization. The beam fully fills the back aperture of a 100 ×, 1.25 NA Nikon E-plan oil immersion objective. From there, the laser passes through the objective, sample, and condenser (10 × objective, Nikon MRP70100). After the condenser, the beam is focused onto the APD. During trapping experiments, the laser is slightly under-focused to maximize transmission to the APD.

### NOT data acquisition and analysis

Data was acquired at a sampling rate of 100 KHz using an Advantech USB-4711A data acquisition unit and analyzed in Python and vbFRET. vbFRET was used for HMM fitting of the data for identifying state transitions. As the program was designed for single-molecule FRET data, input to the program must contain two columns of data corresponding to the donor and acceptor intensity^59^. The FRET efficiency is calculated as ${I}_{{\rm{Eff}}}=\frac{{I}_{A}}{{I}_{D}+{I}_{A}}$. Given that we want to fit the trapping transmission data (*I*_*T*_) as *I*_Eff_, we must have the equivalence of *I*_Eff_ = *I*_*T*_. This is achieved by setting *I*_*T*_ = *I*_*A*_ and *I*_*D*_ = 1 − *I*_*A*_. HMM fitting was applied with the setting of the number of maximum states as 2 and 20 fitting attempts.

After completing the HMM fitting, the fit was verified visually and refined using a custom Python script that corrects rapid state transitions in the time series data. The script identifies binary state values from the HMM fit and applies a 0.05 s duration threshold to detect potential spurious transitions likely caused by noise rather than actual state changes. For each transition detected within or below the threshold, the script compares the mean signal value during the brief state to the midpoint between the high and low states. If the mean value deviates by less than 10% from this midpoint, the transition is considered noise, and the state is reverted to the previous one. This correction process helps to remove spurious transitions, preserving both event duration and the integrity of the fit in the corrected time series. Refer to Supplementary Fig. SI-7 for the results of pre- and post-fitting improvements. Residence times are then collected after a quality check of the fitting.

Once all bound and unbound residence times are collected, we plot residence times as a CDF. This is then fit to the equation of $1-\exp \left(-\frac{x}{\tau }\right)$ using *scipy.optimize.curve_fit*, where *τ* is the time constant. Using equations given by the law of mass action, we calculated the binding rate constants as well as dissociation constant. We do not make the assumption of [*L*]_*total*_ ≈ [*L*]_*free*_ as this would introduce error to *k*_*on*_ and *K*_*D*_ as ligand depletion would remain unaccounted. Using time constants *τ*_*unbound*_ and *τ*_*bound*_ from the CDF fit, we use quadratic Eq. (5) to calculate for the free concentration of SMAP as well as *τ*_*on*_ and *K*_*D*_.

The protein concentration [*P*] and ligand concentration [*L*] bind as:

1 $$[P]+[L]\mathop{\rightleftharpoons }\limits_{{k}_{{\rm{off}}}}^{{{k}_{{\rm{on}}}}}[PL]$$

The on binding rate is given by:

2 $${k}_{{\rm{on}}}=\frac{1}{{\tau }_{{\rm{on}}}\cdot {[L]}_{{\rm{free}}}}$$

and the off binding rate is given by:

3 $${k}_{{\rm{off}}}=\frac{1}{{\tau }_{{\rm{off}}}}$$

From these, the dissociation constant can be determined as:

4 $${K}_{D}=\frac{{\tau }_{{\rm{on}}}\cdot {[L]}_{free}}{{\tau }_{{\rm{off}}}}=\frac{[P][L]}{[PL]}={\tau }^{* }({[L]}_{t}-[PL])=\frac{({[P]}_{t}-[PL])({[L]}_{t}-[PL])}{[PL]}$$

where we have exploited the relation:

5 $$\left({[PL]}^{2}+{\tau }^{* }{[PL]}^{2}\right)-[PL]\cdot \left({[P]}_{t}+{[L]}_{t}+{\tau }^{* }{[L]}_{t}\right)+{[P]}_{t}\cdot {[L]}_{t}=0$$

The Gibbs free energy of association is calculated using this equation.

6 $$\Delta {G}^{\circ }\,=RT\ln {K}_{{\rm{D}}}$$

### Docking simulations

ATUX-8385 was docked to PR65 using ROSIE^43^ and AutoDock Vina^60^. Before initiating the docking simulations using AutoDock Vina, hydrogens were added to both protein and ligand, and the structures were saved in the PDBQT file format utilizing AutoDock Tools (ADT) 1.5.6^46^. Subsequently, partial charges for all ligands were calculated using the Gasteiger method within ADT. The added hydrogens in ligand were manually inspected. Following this verification, a 3-dimensional grid box was constructed with varying sizes ranging from 20 Å to 40 Å, maintaining a grid spacing of 1 Å. The center of the initial grid box was set based on the ATUX-8385 docked structure obtained from ROSIE.

### System preparation for MD simulations

The structure of monomeric PR65 was derived from the DT-061 bound trimeric PP2A structure (PDB: 6NTS^45^) with a resolution of 3.63 Å, in which PR65 adopts a compact form. The coordinates for ATUX-8385 docked at 4_*i*_, 5_*i*_, 5_*o*_, and 6_*o*_ were obtained from our docking simulations. Each of apo and ATUX-8385 bound PR65 structures was solvated in a water box containing explicit TIP3P^61^ water molecules, with a box size 144 Å in all directions. To neutralize the system and set the ion concentration to 150 mM NaCl, Na+ and Cl- ions were added. The system size was approximately 286,900 atoms. All system preparation steps were conducted using VMD^62^.

### Details of MD simulations

MD simulations were conducted using the NAMD 3^63^ software, employing the CHARMM36 all-atom additive protein force field^64^. A 2 fs time step was used throughout the simulations. Temperature was maintained at 310 K via Langevin dynamics, utilizing a damping coefficient of 1 ps-1, and pressure was held at 1 atm using the Langevin Nosé–Hoover method with an oscillation period of 100 fs and a damping time scale of 50 fs. Van der Waals interactions were calculated with a cut-off distance of 12 Å, while the particle-mesh Ewald method was applied for long-range electrostatic interactions. Two rounds of system minimization and equilibration were executed before each production run. Initially, the protein structure was kept fixed and the system was to 10,000 minimization steps, followed by a 1 ns of equilibration. This first round of minimization-equilibration was designed to equilibrate the solvent around the protein. Subsequently, we performed a second round of minimization-equilibration, in which the system underwent an additional 10,000-step minimization without any restrictions on protein structure and dynamics, followed by a 2 ns of equilibration, applying harmonic constraints (k = 1 kcal/mol/Å^2^) only on the C*α* atoms. After these preparatory simulations, we removed all constraints and initiated the production runs. Apo PR65 simulations were extended to a total duration of 704 ns from our previous study^33^, where each simulation was 654 ns in length. In contrast, all system preparation and production runs for ATUX-8385 bound PR65 were entirely performed in this study.

### Nano-differential scanning calorimetry (nanoDSF)

PR65 samples (2 μM) in PBS, 2 mM DTT were mixed with ATUX-8385 to a final concentration of 100 μM, 10% DMSO and in a total volume of 20 μL. The mixtures were allowed to equilibrate at room temperature for 10 min. High-sensitivity capillaries (NanoTemper) were filled with the equilibrated PR65-SMAP mixtures using capillary forces, ensuring no air bubbles were present. The capillaries were loaded into the Prometheus NT.48, and a thermal ramp from 20 °C to 90 °C at a rate of 1 °C per minute was applied. Intrinsic tryptophan and tyrosine fluorescence at 330 nm and 350 nm was continuously monitored. The melting temperature (*T*_*m*_) was determined from the first derivative of the fluorescence ratio (I350/I330) with respect to temperature. The *T*_*m*_ corresponds to the inflection point of the melting curve.

### Fluorescence polarization (FP)

A fixed concentration of ATUX-8385 (2.5 μM, 5 μM, and 10 μM) was mixed with varying concentrations of the protein (0–80 μM) in a total volume of 20 μL in each well of a 384-well microplate (Greiner), to a final DMSO concentration of 10%. The plate was incubated at 40 °C for 30 min to ensure binding equilibrium. Fluorescence polarization was measured at 40 °C using an excitation wavelength of 295 ± 10 nm and an emission wavelength of 360 ± 20 nm, on a CLARIOStar (BMG Labtech) measuring the light in parallel and perpendicular planes relative to the excitation plane. Wells containing only ATUX-8385 (without PR65) and wells containing only PR65 were used as controls to determine background fluorescence and polarization values. Fluorescence polarization (P) was calculated using the equation:

7 $$P=\frac{{I}_{\parallel }-{I}_{\perp }}{{I}_{\parallel }+{I}_{\perp }}$$

where *I*_∥_ and *I*_⊥_ are the intensities of fluorescence parallel and perpendicular to the excitation plane, respectively. Non-linear regression analysis was performed using a one-site binding model to fit the data and determine the dissociation constant *K*_*D*_, using the following equation:

8 $$P=\frac{{P}_{\max }\cdot {C}_{{\rm{PR65}}}}{{k}_{D}+{C}_{{\rm{PR65}}}}$$

where *P* is the polarization, ${P}_{\max }$ is the maximum polarization at the plateau, *C*_PR65_ is the concentration of PR65 (μM) and *K*_*D*_ is the equilibrium dissociation constant.

### ^19^F Nuclear magnetic resonance (NMR)

NMR samples containing 5% *D*_2_*O* were prepared with ATUX-8385 added to a final concentration of 100 μM in 10% DMSO. NMR spectra were recorded at 298 K on a 600 MHz (^1^*H*) Bruker Avance III spectrometer equipped with a 5 mm QCI HFCN/z cryoprobe (^19^*F* 564 MHz) (Department of Biochemistry, University of Cambridge). 1D ^19^*F* NMR data were obtained with 256 scans for ATUX-8385 in the presence of PR65 and 128 scans without the protein and recorded as 1D CPMG experiments (the separation between neighboring 180^∘^ pulses was 100 μs). To assess binding of ATUX-8385 the intensities of pairwise experiments recorded with 2 ms and 102 ms transverse delay time were compared and the intensity ratios were converted into transverse relaxation rate *R*_2_ values.

## Supplementary information

Supplementary Information

## Data Availability

Data and code is available at https://github.com/nanoplasmonics/smaps.
