# Supplementary material for: Direct observation of small molecule activator binding to single PR65 protein
Source: NPJ Biosens. 2025 Jan 16;2(1):2. doi: 10.1038/s44328-024-00018-7 (PMC11738983; doi:10.1038/s44328-024-00018-7)
Supplement: Supplementary file 1 — Supplementary Information [file 44328_2024_18_MOESM1_ESM.pdf]

Supplementary Information: Direct Observation of Small Molecule Activator Binding to Single PR65 Protein

Annie Yang-Schulz[1,+], Maria Zacharopoulou[2,+], Sema Zeynep Yilmaz[3], Anupam Banerjee[4], Satyaki Saha[4,5], Daniel Nietlispach[6], Michael Ohlmeyer[7], Mert Gur[8], Laura S. Itzhaki[2], Ivet Bahar[4,5], and Reuven Gordon[1,*]

[1]Department of Electrical Engineering, University of Victoria, Victoria, V8W 3P6, Canada
[2]Department of Pharmacology, University of Cambridge, Tennis Court Road, Cambridge CB2 1PD, UK.
[3]Department of Mechanical Engineering, Istanbul Technical University, Istanbul, 34437, Türkiye
[4]Laufer Center for Physical and Quantitative Biology, Stony Brook University, NY 11794, USA
[5]Department of Biochemistry and Cell Biology, School of Medicine, Stony Brook University, NY 11794, USA
[6]Department of Biochemistry, University of Cambridge, Tennis Court Road, Cambridge CB2 1QW, UK
[7]Atux Iskay LLC, Plainsboro, New Jersey, NJ 08536, USA.
[8]Department of Computational and Systems Biology, School of Medicine, University of Pittsburgh, Pittsburgh, PA 15213, USA
[*]rgordon@uvic.ca
[+]these authors contributed equally to this work

ABSTRACT

Scanning electron microscope images of double nanoholes, solubility data for ATUX-8385, binding at low concentrations, docking conformations schematic, binding constants for individual proteins trapped and fluorescence of ATUX-8385.

1 PR65 in low concentration of SMAP

Using the NOT technique for the single-molecule binding assay at a reduced ligand concentration of 5 μM results in a lower frequency of binding events and decreased occupancy of the protein's higher scattering, elongated state. As shown in Figure SI-1a, the trapping signal of PR65 protein differs with 20 μM and 5 μM concentrations of ATUX-8385, with the lower concentration exhibiting a prolonged duration in the more compact form. At lower ligand concentration (5μM), the frequency of binding events decreases compared to 20 μM (Figure SI-1b). Specifically, at this reduced concentration (n=4), the protein spends 37% of its time in the elongated state, compared to 52% at 20 µM (n=8) (Figure SI-1c). This difference allows us to identify the bound state as the more elongated form of the protein.

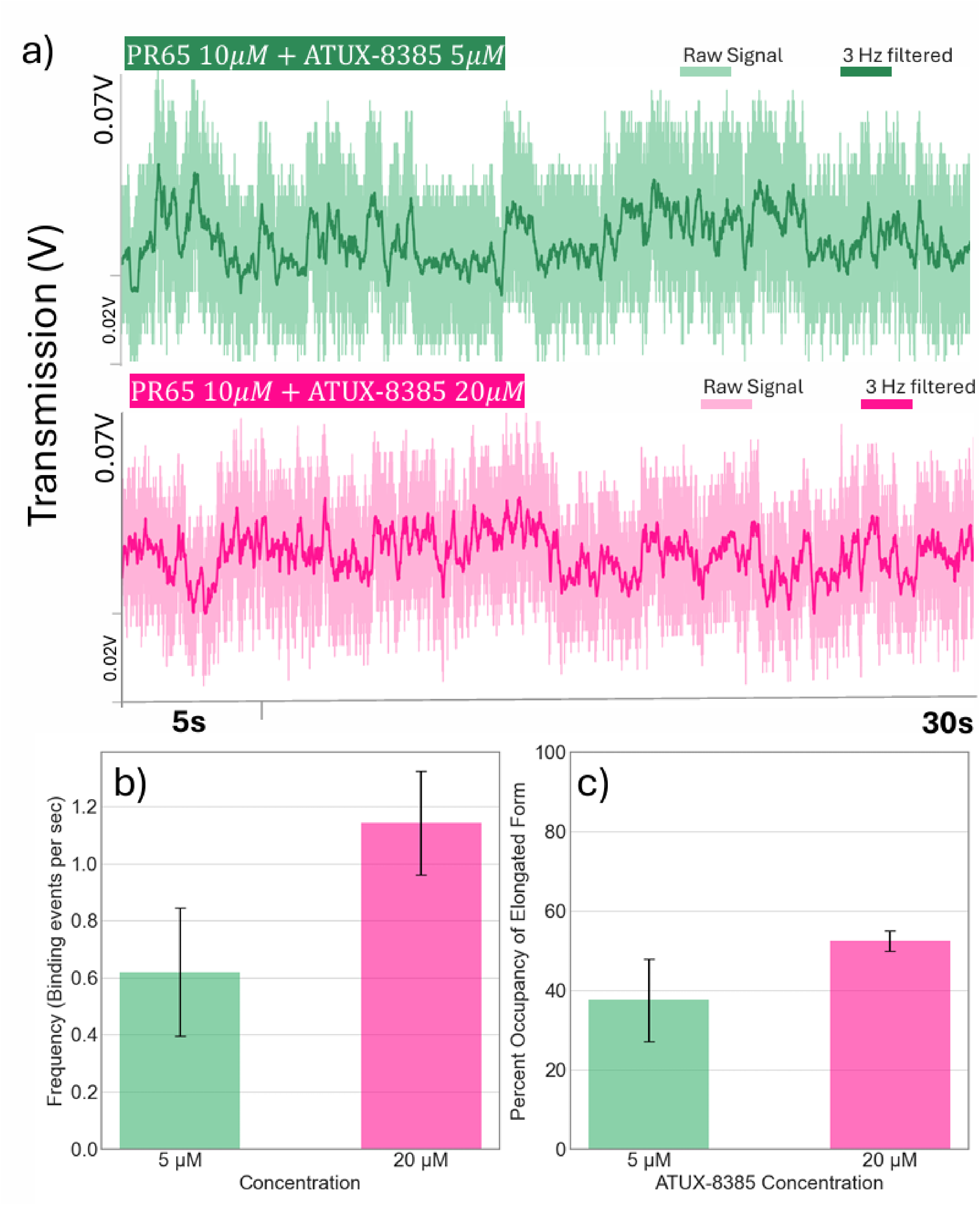

Figure SI-1. **Comparison between low and high ATUX-8385 concentration** (a) Trapping signal of PR65 protein with 20 μM and 5 μM of ATUX-8385. Relative to high concentration of small molecule, the low concentration scattering signal exhibited a prolonged duration in the more compact form. (b) Bar plot comparison of frequency of binding events at a small molecule concentration of 20μM (n=8) and 5μM (n=4). At lower ligand concentration, a decrease in frequency (binding events per second) is observed. (b) At 20μM (n=8) of ligand, the protein on average exist in the elongated form 52% of a time whilst at a ligand concentration of 5μM(n=4), on average the protein exists in elongated form 37% of the time.

2 ATUX-8385 PR65 docked conformation

ATUX-8385 and PR65 protein docked conformations in the compact and extended form are depicted in Figure SI-2.

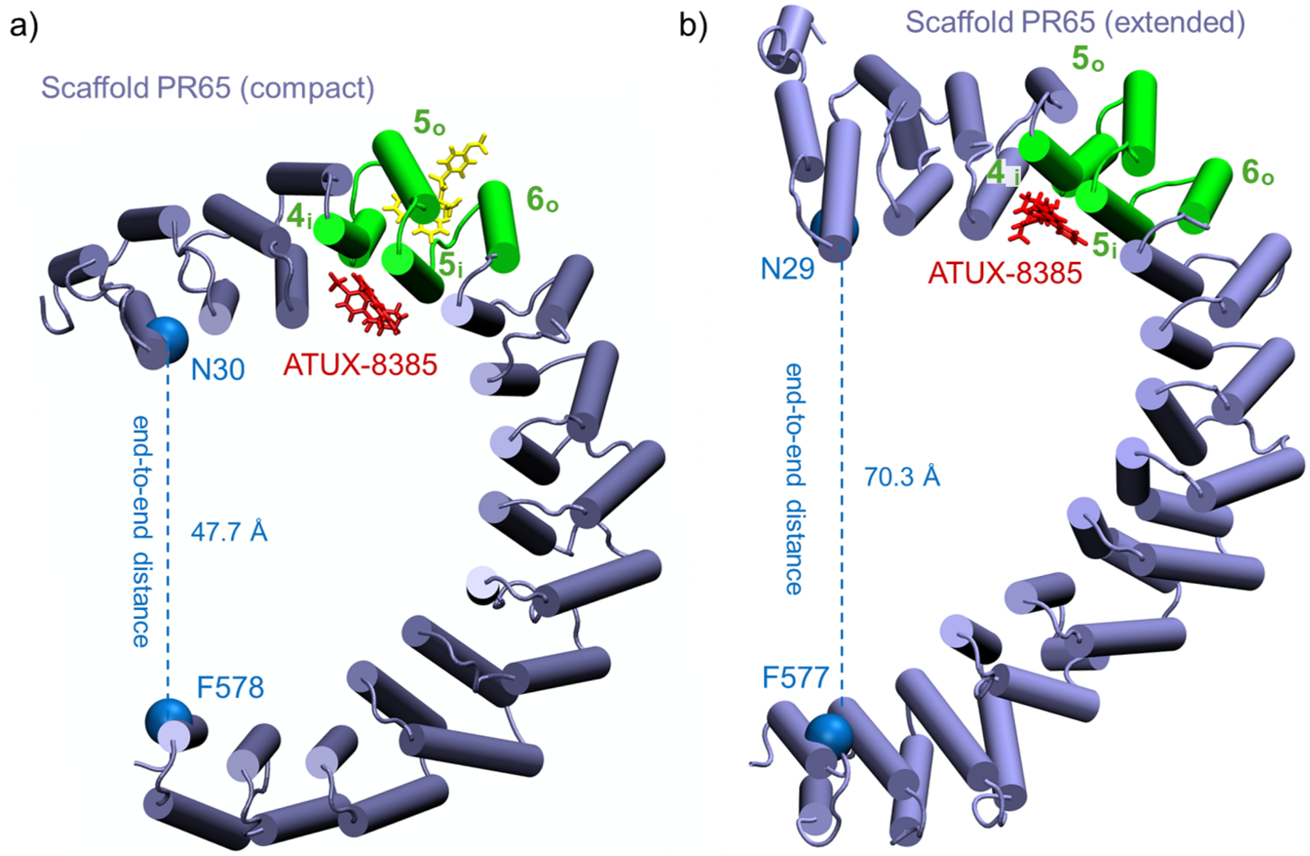

Figure SI-2. ATUX-8385 PR65 docked confirmations (a) ATUX-8385 docked to PR65 in the compact state (PDB: 6NTS): inner helices of HEAT repeats 4 and 5 and outer helices of HEAT repeats 5-6 (yellow, ROSIE) and inner helices of HEAT repeats 4-5 (red, AutoDock Vina). (b) ATUX-8385 docked to PR65 in the extended form(PDB: 1B3U): inner helices of HEAT repeats 4-5 (red, Autodeck Vina)

3 Binding constants of individual proteins

CDF and curve fittings to a single exponential, shown in Figure SI-3, SI-4, of each protein analyzed for Table SI-1.

Table SI-1. Table of ligand dissociation and association rate constants, dissociation constant, coefficient of determination, and duration of the trapped signal analyzed.

**Protein**	k_{off} (s^{-1})	k_{on} ($s^{-1}\mu M^{-1}$)	K_D (μM)	R^2_{Bound}	R^2_{Unbound}	Total time analyzed (s)
PR65 1	2.03	0.16	13.0	0.9885	0.9906	110
PR65 2	2.15	0.19	11.2	0.9834	0.9707	117
PR65 3	2.15	0.17	17.1	0.9838	0.9633	146
PR65 4	2.42	0.20	12.3	0.9884	0.9791	161
PR65 5	2.79	0.19	14.9	0.9749	0.9817	102
PR65 6	3.34	0.19	17.7	0.9625	0.9777	99
PR65 7	2.20	0.19	11.4	0.9769	0.9682	70
PR65 8	2.33	0.21	11.1	0.9636	0.9796	110

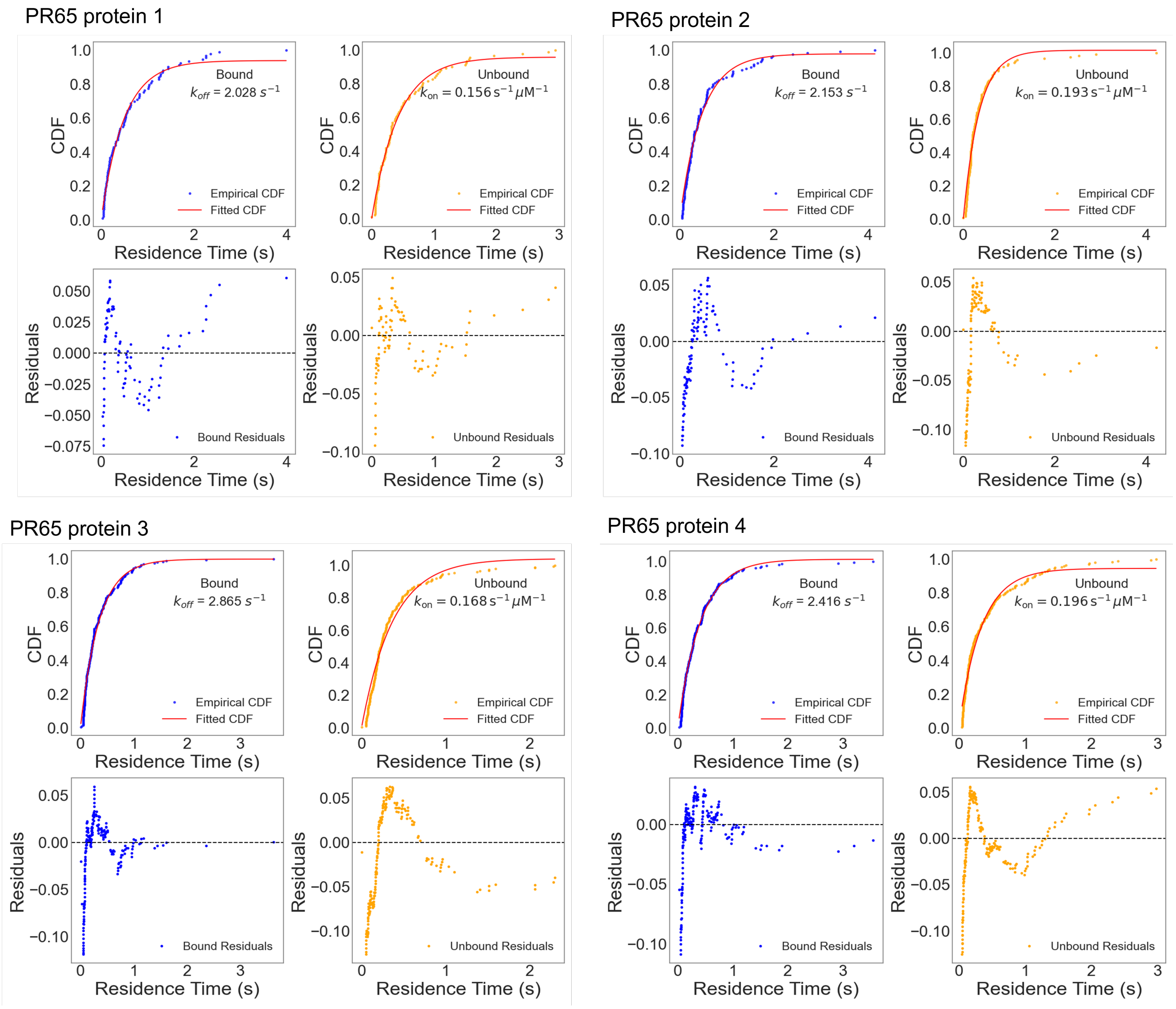

Figure SI-3. CDF curve and single exponential fitting of bound and unbound residence times for protein 1-4.

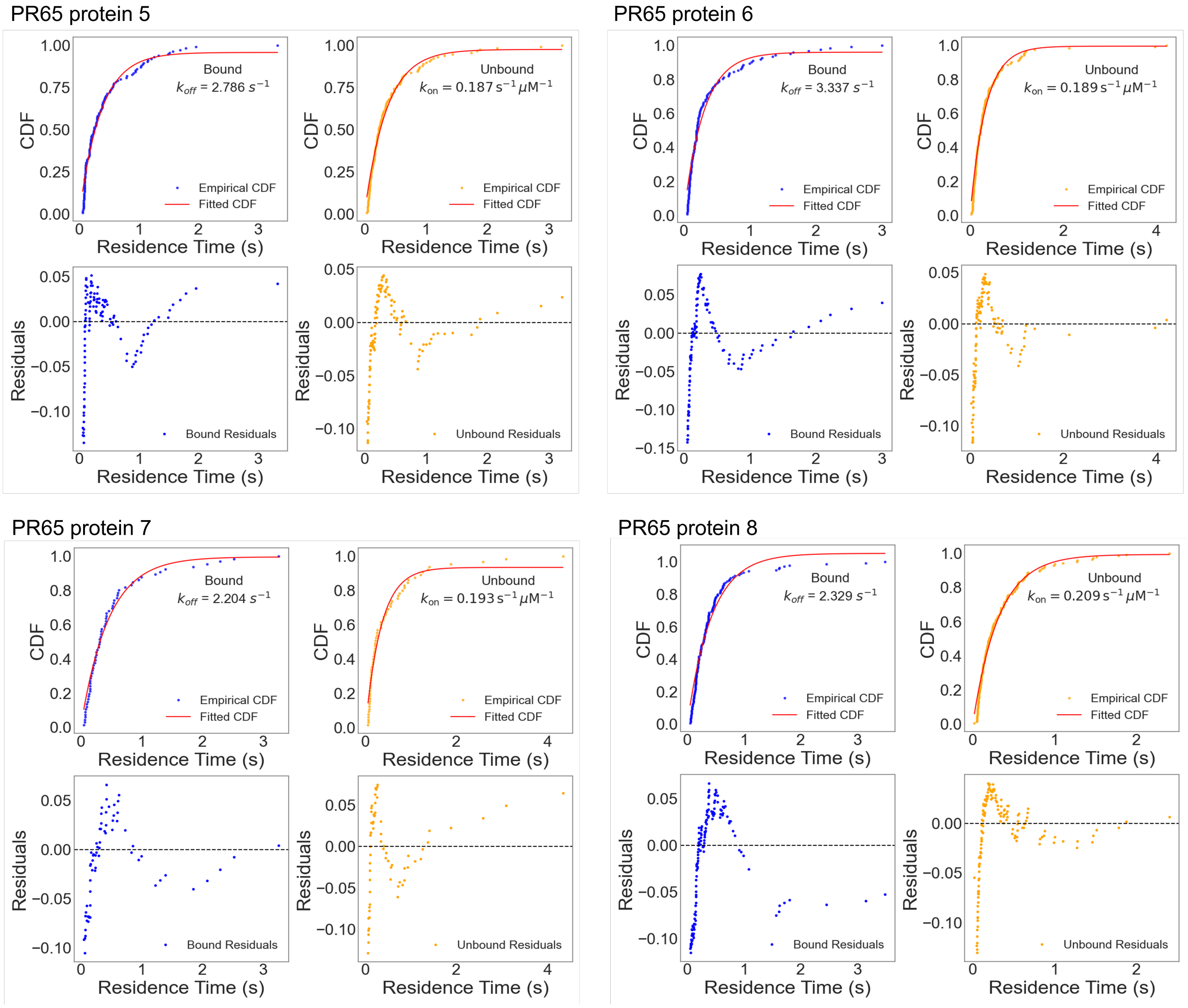

Figure SI-4. CDF curve and single exponential fitting of bound and unbound residence times for protein 5-8.

4 Fluorescence intensity spectra of ATUX-8385

The relatively low solubility of ATUX-8385 SMAP, solutble up to 100 μM in 10% DMSO. Fluorescence Spectra of the small molecule is shown on Figure SI-5.

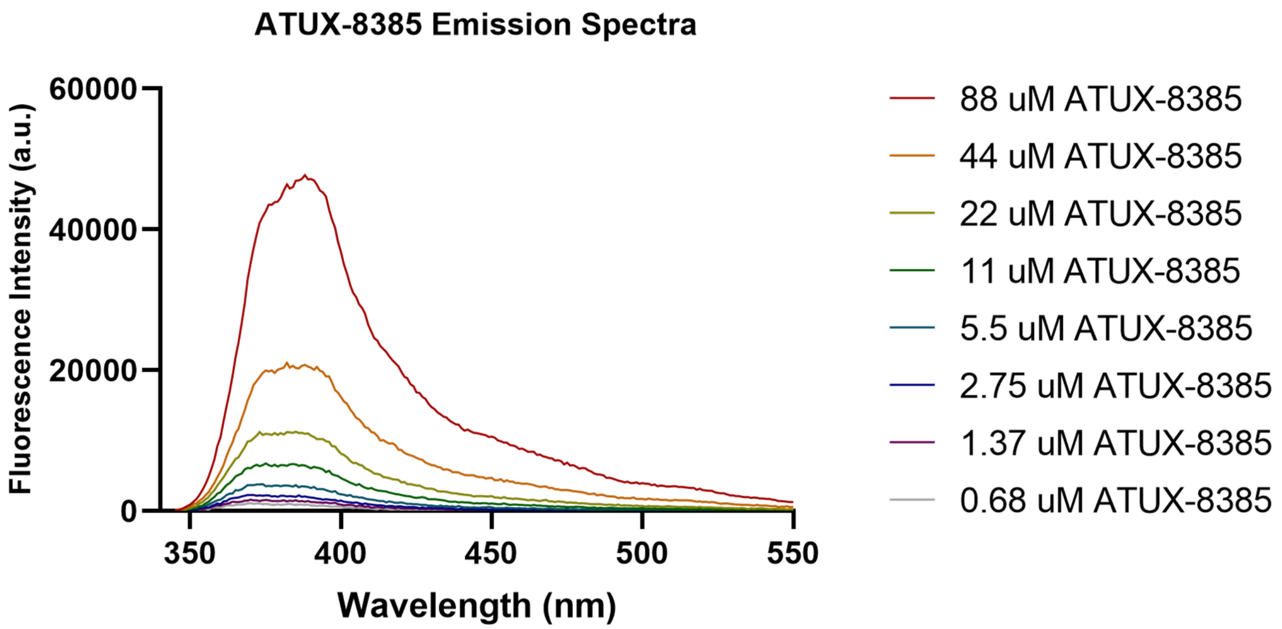

Figure SI-5. Flouresence Intensity Spectra of ATUX-8385. When excited at 290 $\pm$ 10 nm, ATUX-8385 emits from 350 to 550 nm. Samples measured in a 384-well plate, to a final volume of 20 μL and to 10% final DMSO at concentrations from 0.68 μM to 88 μM sequentially diluted.

5 DNHs: SEM images and size distribution

The double-nanohole (DNH) cusp sizes were 62 $\pm$ 16 nm (n=45).

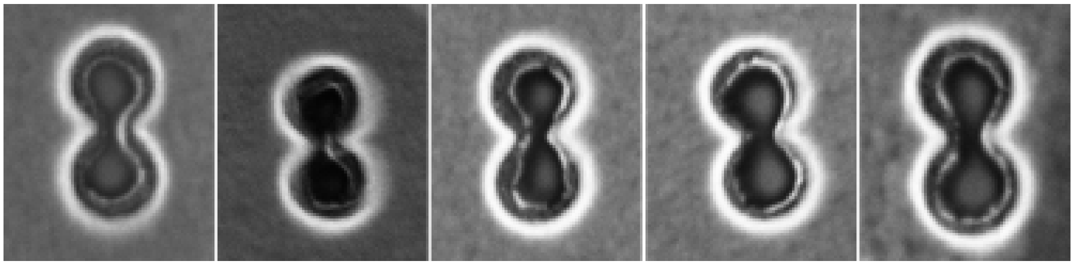

Figure SI-6. SEM images of double-nanoholes.

6 HMM fitting adjustments

As discussed in the methods section, Figure SI-7 provides a sample signal comparing the pre-correction (from vbFRET) and post-correction fitting. The custom Python script used is hosted on GitHub.

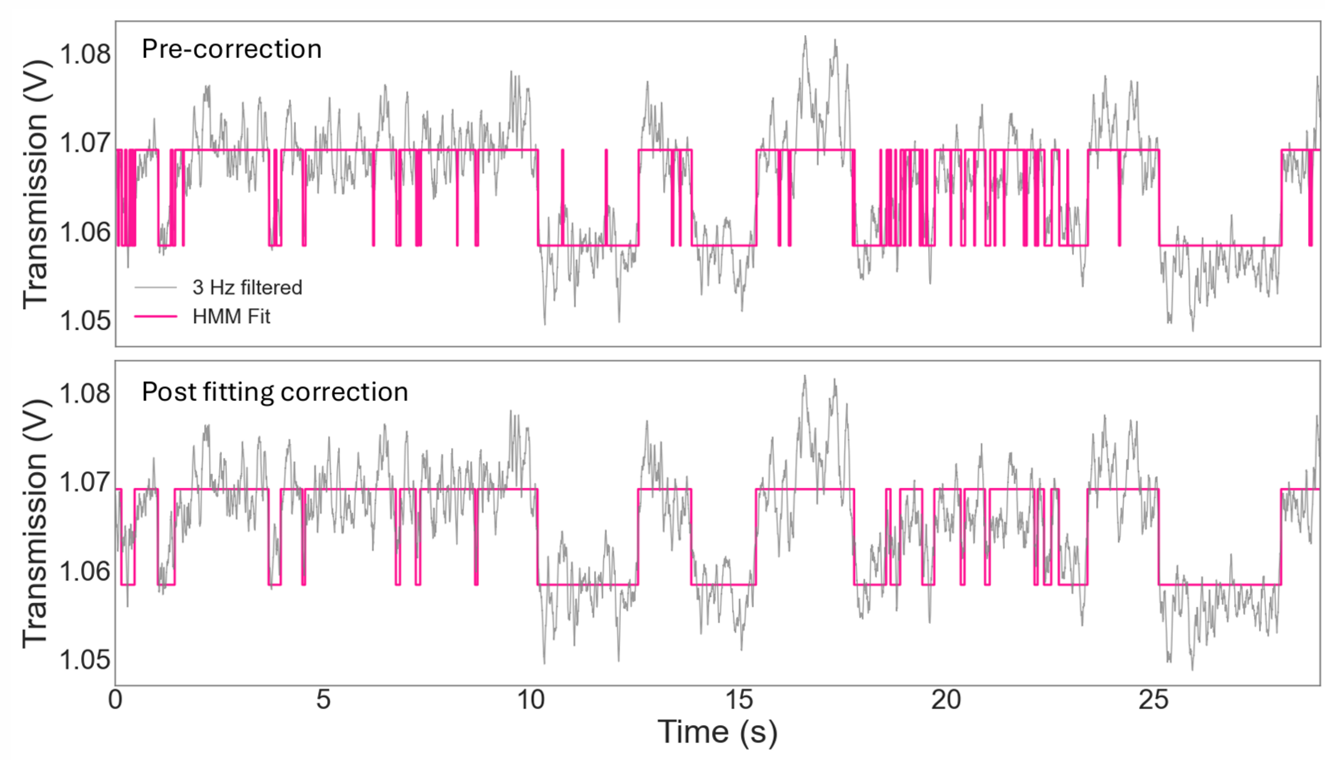

Figure SI-7. Filtered trapped signal showing two state transition with HMM fitting before and after correction. Pre-correction HMM fittings, from vbFRET, shows spurious transitions despite overall decent fitting. Post correction fitting shows a cleaner fit with spurious transitions removed.
